# Calcium Alkynyl(hydrido)zincates

**DOI:** 10.1021/acs.organomet.6c00164

**Published:** 2026-07-09

**Authors:** Marcos López-Aguilar, Kyle G. Pearce, Michael S. Hill

**Affiliations:** a Department of Chemistry, 1555 University of Bath, Claverton Down, Bath BA2 7AY, U.K.; b Organic and Inorganic Chemistry Department, Instituto de Química Organometálica Enrique Moles, Universidad de Oviedo, Oviedo, 33006 Asturias, Spain

## Abstract

The β-diketiminato calcium hydride dimer, [(BDI)­CaH]_2_ (BDI = HC­{(Me)­CNDipp}_2_; Dipp = 2,6-*i*-Pr_2_C_6_H_3_), reacts with [ZnTMP_2_] in the presence of terminal alkynes to provide a structurally diverse series of heterobimetallic calcium–zinc acetylide derivatives. Reactions employing two equivalents of alkyne yield the dialkynyl­(hydrido)­zincates, [(BDI)­Ca­(μ-C≡CR)­(μ-TMP)­Zn­(μ-H)]_2_ (R = *^n^* Bu, Ph), which undergo ligand redistribution to afford the corresponding β-diketiminato calcium acetylides. Conversely, use of four equivalents of alkyne results in further substitution at zinc to provide the tetrakis­(alkynyl)­zincates, [(BDI)­Ca­(μ-C≡C*^n^* Bu)_2_Zn­(μ-H)]_2_ and [(BDI)­Ca­(μ-C≡CPh)_2_Zn­(μ-C≡CPh)_2_Ca­(BDI)]. In contrast, an attempt to synthesize a similarly higher order calcium zincate but containing differentiated alkynyl substituents, by reaction of [(BDI)­Ca­(μ-C≡C*^n^* Bu)­(μ-TMP)­Zn­(μ-H)]_2_ and PhC≡CH, provided [(THF)_2_Ca­(μ-C≡CPh)_3_Zn­(CH_2_Ph)]_2_, resulting from toluene activation and after addition of THF as the only identifiable product.

## Graphical abstract

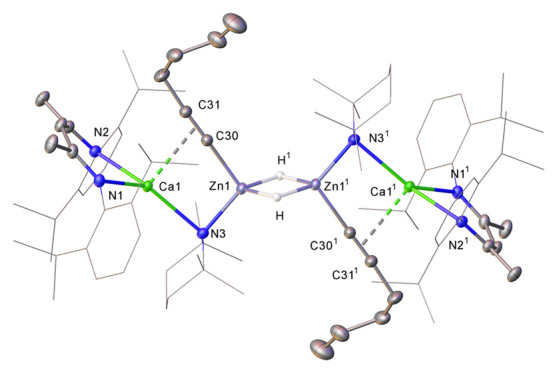

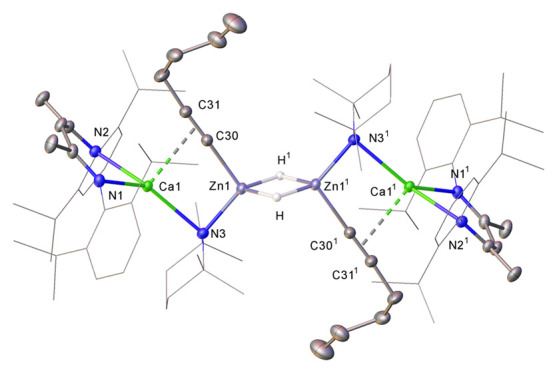

## Introduction

Within the field of *s-*block organometallic chemistry, the use of heterobimetallic reagents is a rapidly evolving area of interest. Such mixed-metal reagents commonly achieve chemical cooperativity, bypassing and surpassing the reactivity profiles of their single metal components. A substantial body of work in this area has now been developed, particularly by the groups of Mulvey and Hevia, the former of whom has even suggested that a ‘Pairiodic Table of the Elements’ may be defined by the synergic reactivity displayed by various combinations of organometallic and metalorganic bases.^1^ These studies have predominantly focused on alkali metal (Li–K) pairings with group 2 metals (notably Mg),^2–13^ as well as *p-*block (B, Al, Ga)^14–17^ and zinc-based partners,^18–23^ establishing a versatile platform for cooperative C–H activation,^24–29^ transmetalation processes,^30–33^ and bimetallic catalysis.^34–43^

Our own earlier interests were focused primarily on the development of highly reactive molecular organocalcium reagents,^44^ initially through reaction of the base-free calcium hydride [(BDI)­CaH]_2_ (**1**; BDI = HC­{(Me)­CNDipp}_2_, where Dipp = 2,6-*i*-Pr_2_C_6_H_3_) with a range of unactivated terminal alkenes.^45–48^ Although the resultant dimeric σ-*n*-alkyl derivatives are sufficiently nucleophilic to even alkylate benzene, this alkene-insertion-based approach is inapplicable to the synthesis of calcium-aryl analogues. Therefore, we recently developed a transmetalation-based strategy to prepare coordinatively unsaturated σ-aryl calcium complexes, [(BDI)­Ca­(μ-H)­(μ-Ar)­Ca­(BDI)] and [(BDI)­Ca­(μ-Ar)­(μ-Ar′)­Ca­(BDI)] (Ar = C_6_H_5_, *ortho*-Me-C_6_H_5_, *meta*-Me-C_6_H_5_, *para*-Me-C_6_H_5_, 3,5-(*t*Bu)_2_C_6_H_3_, Ar′ = C_6_H_5_, *ortho*-Me-C_6_H_5_, *meta*-Me-C_6_H_5_, *para*-Me-C_6_H_5_),^49,50^ through sequential reactions with arylmercuric reagents (Ar_2_Hg; Scheme 1). While unreactive toward benzene, these β-diketiminato arylcalcium derivatives facilitate uncatalyzed access to biaryl molecules by direct S_N_Ar displacement of halide from aryl bromides.

**Scheme 1:**
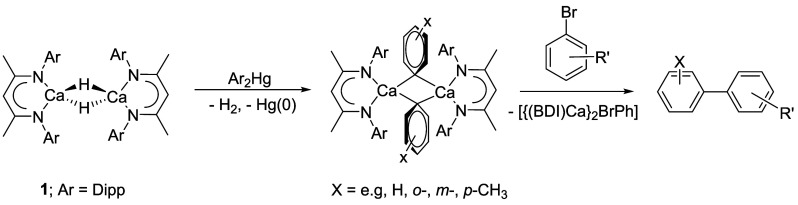
Use of Compound **1** in the Synthesis of Arylcalcium Compounds and Further Reactivity with Aryl Bromides to Prepare Biaryl Molecules

Avoiding toxic organomercurials, a similar transmetallative route can be achieved with dialkyl zinc reagents facilitating access to calcium alkyls including the simplest methylcalcium homologue, [(BDI)­CaMe]_2_.^51,52^ In contrast to mercury, however, the greater stability of zinc hydrides results in a more complex synthetic pathway, proceeding in a stepwise manner via the initial formation of dialkyl­(hydrido)­zincate intermediates (**2a**–**c**; Scheme 2). Although these species undergo subsequent intramolecular equilibration to yield the β-diketiminato calcium alkyl complexes, if retained in solution with [ZnH­(alkyl)]_n_ further intermolecular transmetalation affords the β-diketiminato zinc alkyls, which is irreversible due to the insoluble polymeric nature of [Ca­(H)­(alkyl)]_∞_.

**Scheme 2:**
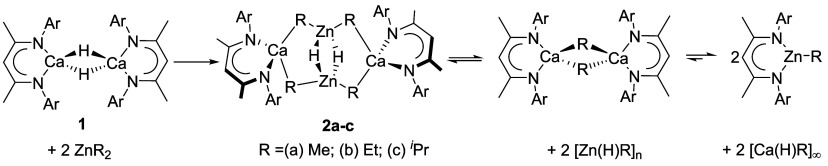
Reaction Pathway between ZnR_2_ (R = (a) Me, (b) Et, (c) ^i^Pr) and [(BDI)­CaH]_2_ (**1**) at Ambient Temperature

We have also recently extended this zinc-based strategy to heteroatomic anions,^53,54^ observing that similar equilibria are established from treatment of [(BDI)­CaH]_2_ with the zinc amides, [Zn­{N­(SiMe_3_)_2_}_2_] and [ZnTMP_2_] (TMP = 2,2,6,6-tetramethylpiperidide). The related calcium amido­(hydrido)­zincates, [(BDI)­Ca­{N­(SiMe_3_)_2_}­(μ-H)­Zn]_2_ and [(BDI)­Ca­(μ-N­{C­(CH_3_)_2_CH_2_}_2_CH)­(μ-H)­Zn­(μ-H)]_2_ (**3**) were identified along with the ensuing respective calcium amides, [(BDI)­Ca­{N­(SiMe_3_)_2_}] and [(BDI)­Ca­(TMP)]. In contrast to the alkylzinc-derived systems, however, the reaction with [Zn­{N­(SiMe_3_)_2_}_2_] ultimately resulted in exchange of both the hydride and BDI ligands from calcium to zinc, affording [(BDI)­ZnH] (Scheme 3a). More significantly, the reaction of [ZnTMP_2_] with **1** in arene solvents, evokes a synergistic partnership between calcium and zinc to enable a sequence of *sp* ^*2*^ C–H deprotonation followed by ligand transmetalation to ultimately afford the corresponding β-diketiminato zinc aryls, which can be directly exploited in Negishi biaryl coupling reactions (Scheme 3b).

**Scheme 3:**
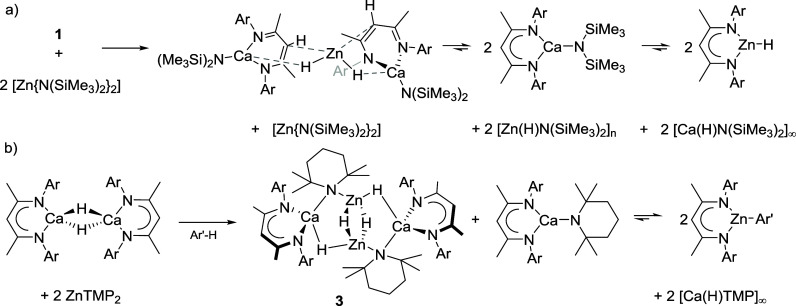
Reaction Pathway Arising from the Reaction of [(BDI)­CaH]_2_ (**1**) and (a) [Zn­{N­(SiMe_3_)_2_}_2_] or (b) [ZnTMP_2_]

In this contribution, we report a continuation of this work, exploring the reactivity of the calcium/zinc bimetallic partnership toward the more readily activated *sp* C–H bonds of terminal alkynes (p*K*_a_ ∼ 24).^55^ This reactivity leads to the formation of dialkynyl­(hydrido)­zincates and tetrakis­(alkynyl)­zincates, which ultimately equilibrate to the corresponding β-diketiminato calcium acetylides.

## Results and Discussion

An initial reaction of two equivalents of 1-hexyne in *d*_8_-toluene with a mixture of compound **1** and two equivalents of [ZnTMP_2_] was observed to result in the immediate consumption of the calcium and zinc reagents by ^1^H NMR spectroscopy. The resulting spectrum evidenced a single BDI containing species (**4**), exhibiting a characteristic BDI γ-methine singlet at δ_H_ 4.76 ppm, as well as two associated triplet resonances, observed at 2.40 and 0.94 ppm and which integrated in a respective 2:3 ratio relative to the 1H BDI γ-methine signal. The latter resonances were assigned to the respective methylene and methyl environments positioned α- and δ- to the triple bond of a newly formed hexynyl anion, while a further (1H) singlet observed at 3.80 ppm was tentatively attributed to a bridging Zn–H environment by comparison to the analogous signals previously assigned for the zincate species [(BDI)­Ca­(μ-N­{C­(CH_3_)_2_CH_2_}_2_CH)­(μ-H)­Zn­(μ-H)]_2_ (**3**; 5.01 ppm), and [(BDI)­Ca­(μ-R)_2_Zn­(μ-H)]_2_ (**2a**–**c**; R = (**a**) Me; (**b**) Et; (**c**) *^i^* Pr; 3.06–3.15).^51,52^ The apparent retention of the TMP, alkyne and hydride functionalities, thus, implies the formation of a similar bimetallic zincate structure, an inference subsequently confirmed by single crystal X-ray diffraction analysis (Figure 1).

**Figure 1:**
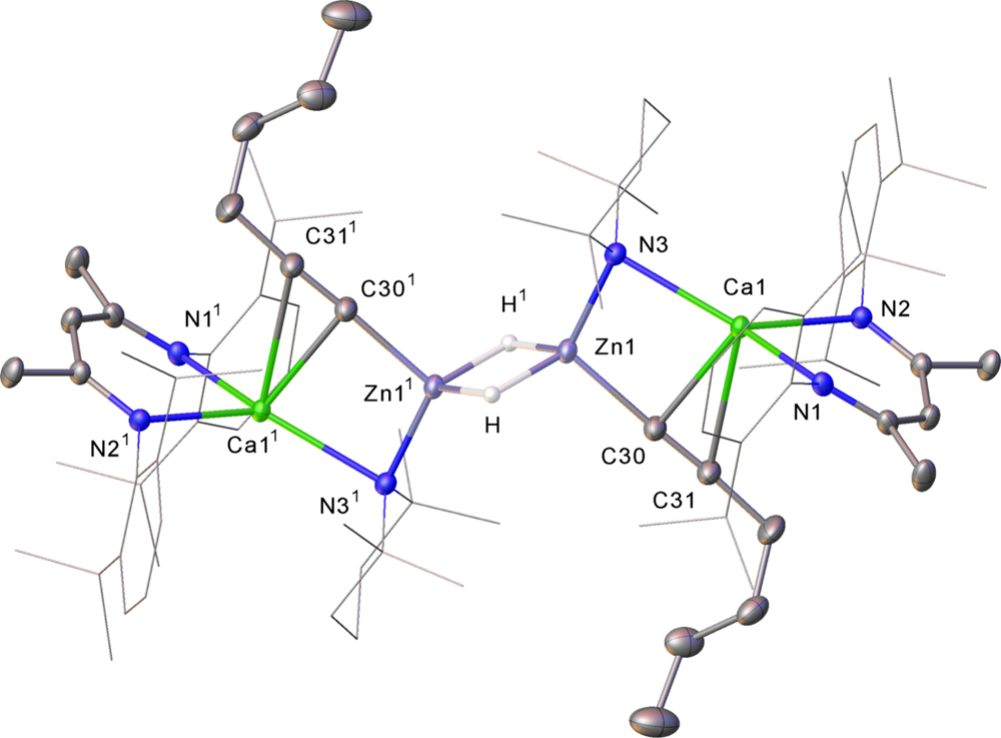
Molecular structure of **4**, displacement ellipsoids at 30%. For clarity, hydrogen atoms, apart from the bridging hydrides are omitted. Dipp and TMP groups are displayed as wireframe, also for visual ease. Selected bond lengths (Å): Ca1–N1 2.3657(13), Ca1–N2 2.3614(12), Ca1–N3 2.4771(13), Ca1–Zn1 3.1023(5), Ca1–C30 2.5131(16), Ca1–C31 2.8415(18), Zn1–N3 2.0217(13), Zn1–C30 2.0217(13), Zn1–H 1.686(17), Zn1–H^1^ 1.699(18), Zn1–Zn1^1^ 2.4382(7). Symmetry operations to generate primed atoms: ^1^1 – x, 1 – y, 1 – z.

Compound **4** was identified as the centrosymmetric dimer, [(BDI)­Ca­(μ-C≡C*^n^* Bu)­(μ-TMP)­Zn­(μ-H)]_2_, in which each Ca atom is coordinated by three *N*-donor contacts, two from each BDI ligand [2.3657(13), 2.3614(12) Å], and the third from a bridging TMP anion between Ca1 and Zn1, [Ca1–N3:2.4771(13)]. This latter bond length is somewhat reduced relative to its closest comparator **3**, [2.5271(14) Å]. The μ_2_-hydride-bridged zinc centers are further coordinated by the TMP nitrogen [Zn1–N3:2.0217(13) Å] and a σ-bonded interaction to the hexynyl anion, the length of which [Zn1–C30 2.0217(13) Å] is consistent with a Zn–C single bond.^56^ The acetylide unit, thus, interacts with calcium [2.5098 (18) Å] via η^2^ engagement of its C≡C bond. Furthermore, in contrast to the precedented zincates illustrated in Schemes 2 and 3, the two μ-H units are exclusively shared between the Zn centers. Although the resultant Zn–H bond distances [1.686(17) and 1.699(18) Å] are intermediate between those of [(BDI)­Ca­{N­(SiMe_3_)_2_}­(μ-H)­Zn]_2_ [1.603(18) Å] and compounds **2a**–**c** and **3** (*ca.* 1.74–1.79 Å), they lie within the range established for precedented bridging zinc hydrides (1.6–1.8 Å).^57–61^

As previously observed for compounds **2a**–**c** and **3**, redissolution of **4** induced further redistribution, affording a solution that presented a ^1^H NMR spectrum consistent with the formation of [(BDI)­Ca­(μ-C≡C*^n^* Bu)]_2_ (**5**),^62^ which was clearly identified by its characteristic CH_2_C≡C triplet (1.87 ppm) and BDI γ-methine singlet (4.71 ppm) resonances (Figure S3) and confirmed by X-ray diffraction analysis (Figure 2). It is worth noting that **5** can readily be formed through the deprotonation of 1-hexyne by compound **1**.^62^ In contrast to the precedented calcium zincate chemistry (Schemes 1 and 2), no evidence for further transmetalation of the BDI or acetylide anions to zinc was observed and the reaction terminated after the formation of compound **5** (Scheme 4). This behavior presumably reflects the more robust nature of the dimeric acetylide resulting from the greater coordinative saturation provided by the combination of σ- and π-interactions provided by the organic anions to each calcium.

**Figure 2:**
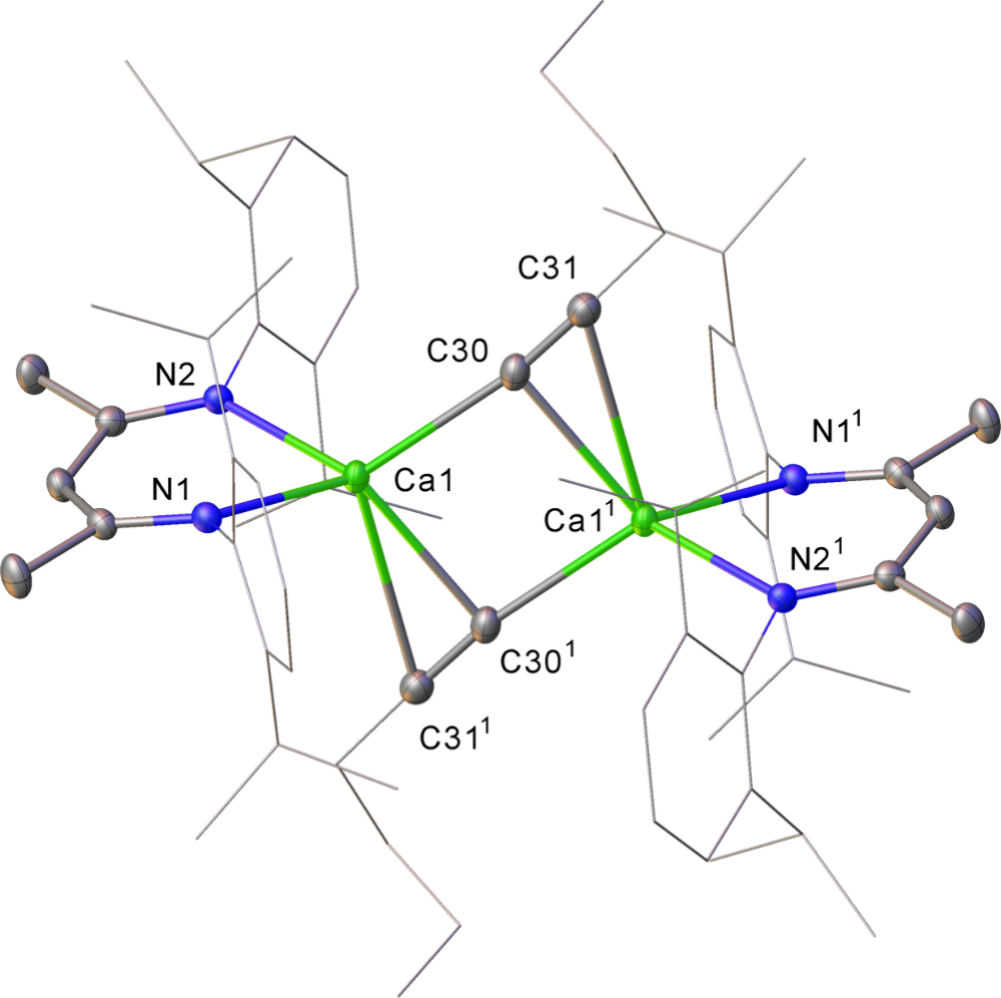
Molecular structure of **5**, displacement ellipsoids at 30%. For clarity, hydrogen atoms have been omitted. Dipp substituents and the alkyne chain apart from C30 and C31 are displayed as wireframe. Selected bond lengths (Å): Ca1–N1 2.3145(11), Ca1–N2 2.3266(11), Ca1–C30 2.4852(15), Ca1–C31 3.7075(19), Ca1–C30^1^ 2.5116(15), Ca1–C31^1^ 2.9506(16). Symmetry operations to generate primed atoms: ^1^1 - x, 1 - y, 1 - z; ^2^2 - x, 2 - y, 2 - z.

**Scheme 4:**
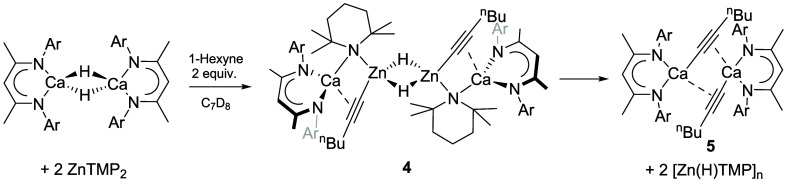
Proposed Reaction Pathway between ZnTMP_2_ and [(BDI)­CaH]_2_ with 1-Hexyne

To assess the generality of this reactivity, *d*_8_-toluene solutions of [ZnTMP_2_] and **1** were treated with two equivalents of 2-ethynylpyridine or phenylacetylene (Scheme 5). In both cases almost immediate crystallization was observed allowing the structural identification of the respective β-diketiminato calcium acetylides, [(BDI)­Ca­(μ-C≡CPy)]_2_ (**6**; Figure 3; py = pyridine) and the previously reported [(BDI)­Ca­(μ-C≡CPh)]_2_,^62^ by X-ray diffraction analysis. The structure of **6** is largely consistent with precedented calcium acetylides, albeit the nitrogen donor from the pyridyl ring acts as an anchor to the calcium atom imposing an acute angle of 11.96° between the plane defined by N3–Ca1 unit and the pyridyl motif. As previously documented,^62^ [(BDI)­Ca­(μ-C≡CPh)]_2_ is insoluble in noncoordinating solvents and could only be identified by its characterization in the solid-state. In contrast, compound **6** could be redissolved in *d*_8_-toluene (Figure S8) with sonication. The pyridyl unit was, thus, readily identified in the resultant ^1^H NMR spectrum, displaying a characteristic downfield 1H resonance at δ_H_ 8.60 and with two further aromatic resonances at 6.81 and 6.40 ppm, accounting for 2 and 1 protons respectively by relative integration. The BDI γ-methine singlet was visible at 4.84 ppm, alongside the accompanying Dipp *iso*-propyl methine resonances, which resolved as two inequivalent broad 2H signals (3.62, 2.67 ppm).

**Scheme 5:**
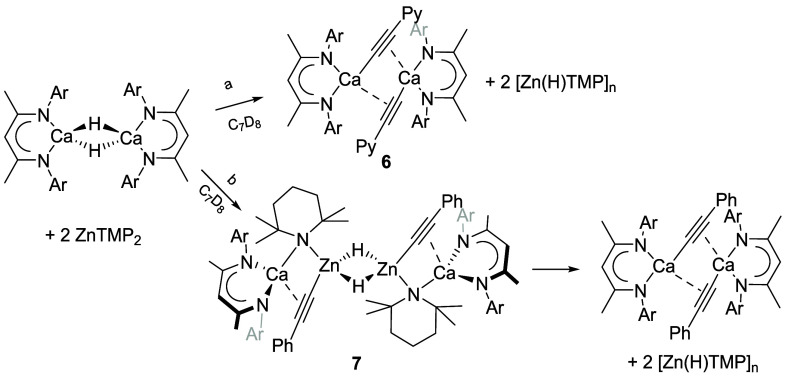
Proposed Reaction Pathway between [ZnTMP_2_] and [(BDI)­CaH]_2_ (**1**) with (a) 2-Ethynylpyridine (py = pyridine) and (b) Phenylacetylene

**Figure 3:**
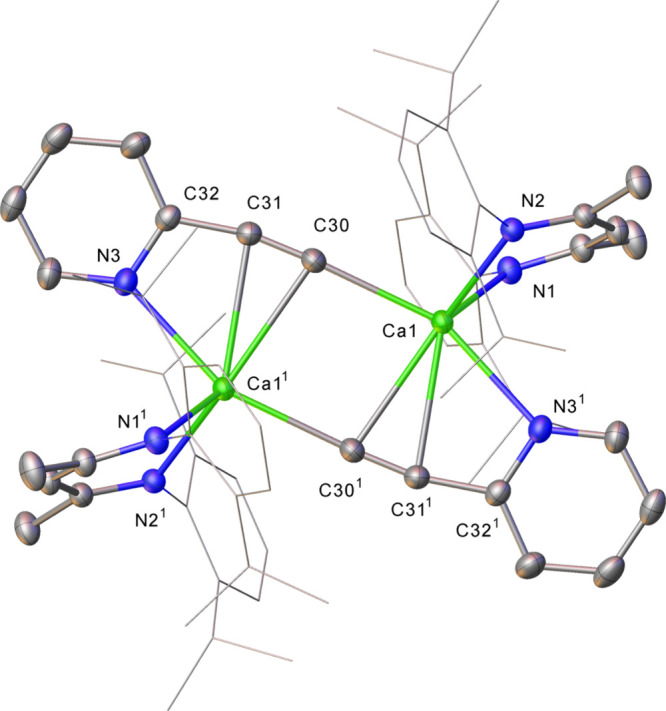
Molecular structure of **6**, displacement ellipsoids at 30%. For clarity, hydrogen atoms have been omitted, and the Dipp units are displayed as wireframe. Selected bond lengths (Å): Ca1–N1 2.3326(12), Ca1–N2 2.3219(11), Ca1–N3 2.5506(13), Ca1–C30 2.4852(16) Ca1–C31 3.7067(16), Ca1–C30^1^ 2.7325(15), Ca1–C31^1^ 2.7411(15). Symmetry operations to generate primed atoms: ^1^1 – x, 1 – y, 1 – z.

In a further attempt to trap and elucidate the structures of any pretransmetalation intermediates, the reactions between **1** and both 2-ethynylpyridine and phenylacetylene were repeated and immediately placed into the freezer (−35 °C). Although treatment of **1** with 2-ethynylpyridine again yielded **6** as the sole identifiable product, the reaction with phenylacetylene provided single crystals of the dialkynyl­(hydrido)­zincate, [(BDI)­Ca­(μ-C≡CPh)­(μ-TMP)­Zn­(μ-H)]_2_ (**7**), which was identified by X-ray diffraction analysis (Figure 4). Compound **7** is a further centrosymmetric dimer with a gross structure analogous to that of compound **4**. Irrespective of the differing alkyne substituents, the primary metric data provided by complexes **4** and **7** are essentially identical, albeit the Zn–H bond in **7** is somewhat elongated relative to **4** [1.81(3) versus 1.693(12) Å].

**Figure 4:**
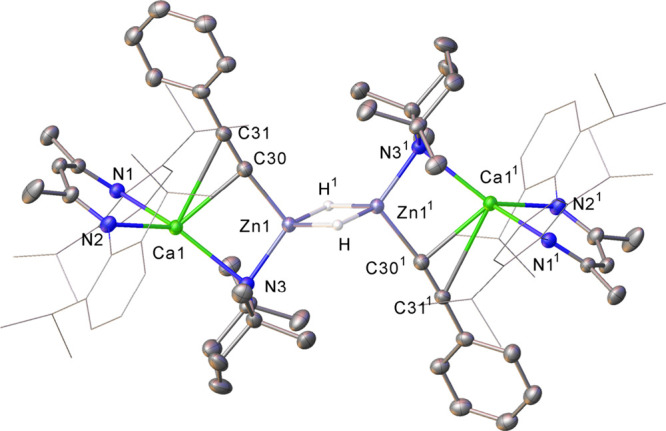
Molecular structure of **7**, displacement ellipsoids at 30%. For clarity, hydrogen atoms, apart from the bridging hydrides are omitted. Dipp groups are displayed as wireframe, also for visual ease and the toluene molecule has been omitted. Selected bond lengths (Å): Ca1–N1 2.392(3), Ca1–N2 2.376(3), Ca1–N3 2.508(3), Ca1–Zn1 3.1067(7), Ca1–C30 2.535(4), Ca1–C313.052(4), Zn1–N3 2.026(3), Zn1–C30 2.028(4), Zn1–H 1.83(4), Zn1–H^1^ 1.79(4), Zn1–Zn1^1^ 2.4540(8).

The retention of two potentially highly basic TMP residues in both **4** and **7** (Schemes 4 and 5; *vide supra*) suggested that further alkyne deprotonation may be achieved. Four equivalents of 1-hexyne or phenylacetylene were, therefore, added to *d*_8_-toluene solutions of compound **1** and [ZnTMP_2_] and immediately placed in the freezer (−35 °C) to afford crops of colorless crystals of compounds **8** and **9**. Although redissolution of both species invariably resulted in the formation of their respective β-diketiminato calcium acetylide dimers, single crystal X-ray diffraction analysis of the crystals themselves elucidated their structures as the tetrakis­(alkynyl)­zincates, [(BDI)­Ca­(μ-C≡C*^n^* Bu)_2_Zn­(μ-H)]_2_ (**8**; Figure 5a) and [(BDI)­Ca­(μ-C≡CPh)_2_Zn­(μ-C≡CPh)_2_Ca­(BDI)] (**9**; Figure 5b).

**Figure 5:**
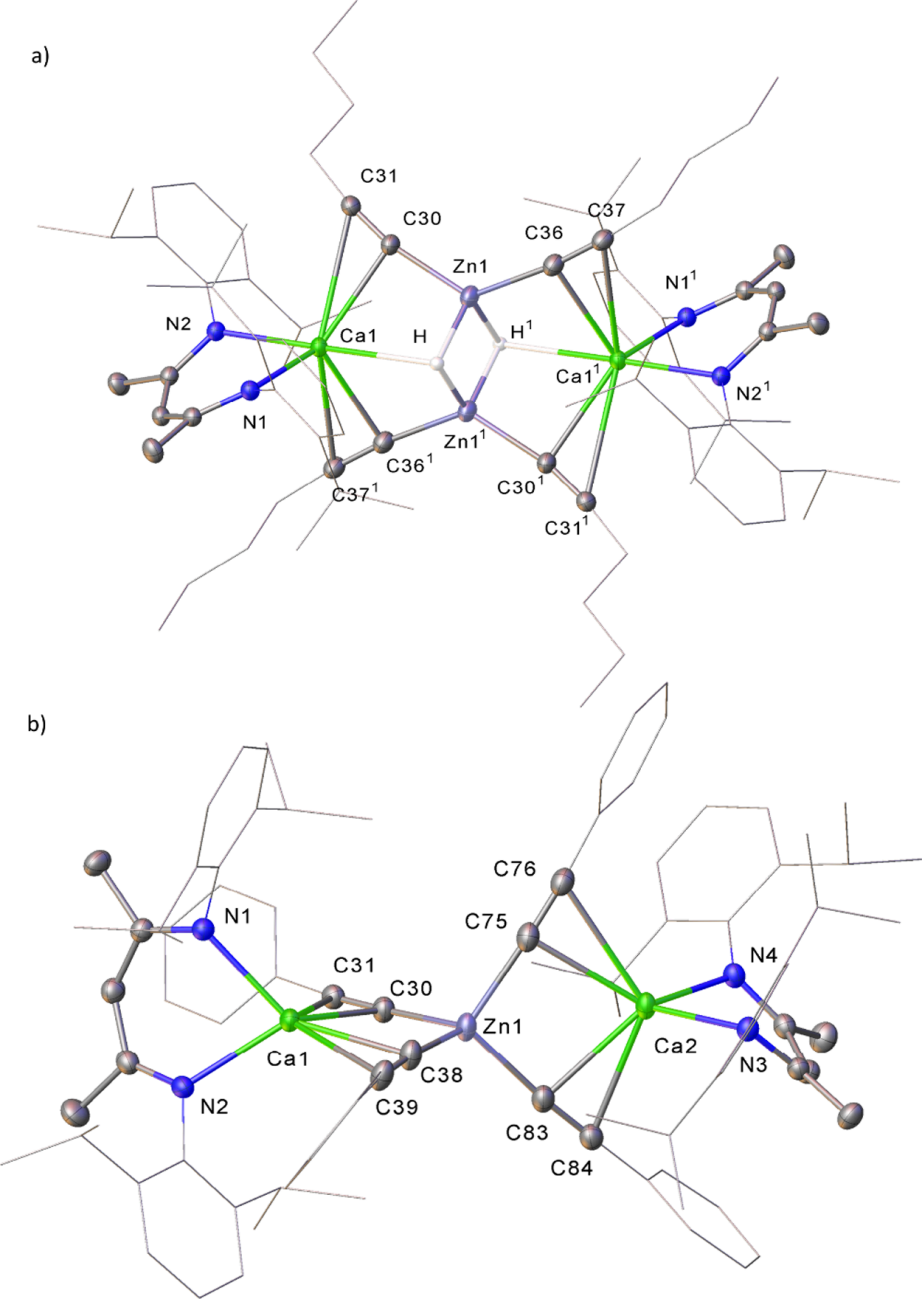
Molecular structures of (a) compound **8** and (b) compound **9** with displacement ellipsoids at 30%. For clarity hydrogen atoms, apart from bridging hydrides have been omitted. Dipp groups, and the alkyne groups apart from C30,C31,C36,C37,C38,C39,C75,C76,C83,C84 have been displayed as wireframe for visual ease. Selected bond lengths (Å): (**8**) Ca1–N1 2.3480(17), Ca1–N2 2.3414(17), Ca1–Zn1 3.2535(4), Ca1–C30 2.613(2), Ca1–C31 3.090(2), Ca1–H1^1^ 3.871(18), Ca1–H 2.830(18), Zn1–C30 1.980(2), Zn1–C36 1.964(2), Zn1–H1^1^ 2.155(18), Zn1–H 1.876(18), Zn1–Zn1^1^ 2.5644(5). Symmetry operations to generate primed atoms:^1^1 – x, 1 – y, 1 – z. (**9**) Ca1–N1 2.3179(14), Ca1–N2 2.3098(14), Ca1–Zn1 3.2046(4), Ca2–Zn1 3.2391(4), Ca1–C30 2.5532(18), Ca1–C31 2.8388(18), Ca1–C38 2.5423(19), Ca1–C39 2.940(2), Zn1–C30 2.0408(19), Zn1–C38 2.052(2), Zn1–C75 2.052(2), Zn1–C83 2.038(2), Ca2–C75 2.5304(19), Ca2–C76 2.849(2), Ca2–C83 2.5880(19), Ca2–C84 2.8192(19).

Although compound **8** provides a further example of a calcium dialkynyl­(hydrido)­zincate dimer, its structure presents a significant contrast to the exclusively Zn-μ-H_2_–Zn bridging identified for both **4** and **7** and is more reminiscent of the dialkyl­(hydrido)­zincates (**2a**–**c**) in which the bridging hydrides interact with both zinc and calcium. A similar asymmetry with respect to the Ca–H interactions is also observed, one elongated by ca. 1 Å relative to the other, [**8**: Ca1–H1 2.830(18) vs Ca1–H^1^ 3.871(18); **2a**–**c**: Ca1–H1 2.35–2.37 vs Ca1–H^1^ 3.40–3.52 Å]. In a similar manner to **4** and **7**, however, the π-component of the alkyne continues to interact exclusively with calcium, albeit with a slight elongation in the relevant Ca–C distances observed for **8** [**8**: Ca1–C30 2.613(2); Ca1–C31 3.090(2); **4**: Ca1–C30 2.5131(16); Ca1–C31 2.8415(18) Å].

In contrast to compound **8**, the dimeric structure of **9** comprises no hydrides and possesses a single Zn center, which is σ-bonded to four phenylacetylide units in a distorted tetrahedral geometry. The resultant tetrakis­(alkynyl)­zincate dianion encapsulates two [(BDI)­Ca] moieties, each via 2-fold η^2^-π-C≡C interactions [Ca···C≡C_centroid_ 2.4483(10) Å] with the group 2 cations. Although, as a likely consequence, the symmetry-related Ca centers are situated 1.072 Å above the least-squares plane defined by the BDI chelate ligands, the Zn–C bond distances in **9** are closely comparable to those of [(PhC≡C)_4_Zn­(Li­(TMEDA)}_2_] (TMEDA = *N,N,N*′*,N*′-tetramethylethylenediamine) containing the identical zincate dianion.^63^

With complexes **8** and **9** in hand, we sought to prepare a tetrakis­(alkynyl)­zincate with differentiated alkyne substituents. Two equivalents of phenylacetylene were, thus, added to a *d*_8_-toluene solution of **4** resulting in immediate precipitation of an insoluble amorphous solid. Treatment of this unidentified material with a few drops of THF led to its dissolution and immediate precipitation of a microcrystalline solid, which was characterized by single crystal X-ray diffraction as the trisalkynyl­(benzyl)­zincate species, [(THF)_2_Ca­(μ-C≡CPh)_3_Zn­(CH_2_Ph)]_2_ (**10**; Figure 6). Compound **10** crystallizes as a further higher order calcium tetraorganozincate, albeit the constitution of the compound now requires two zinc anions to balance the net 4+ charge of the two calcium dications, which are no longer coordinated by BDI ligands. Each calcium center exhibits a *pseudo*-octahedral geometry, with two *cis*-ligated molecules of THF and two η^2^-interactions to each trisalkynylzincate dianion. While the C8–C9 and C24–C25 triple bonds each display an η^2^-engagement with a single Ca cation [Ca···C≡C_centroid_ 2.598(7), 2.646(6) Å], the C16–C17 triple bond bridges both symmetry-related group 2 centers. This interaction has a notable influence on the associated Zn–C bond distance, which is elongated [2.129 (5) Å] in comparison to the corresponding bonds from Zn1 to C8 and C24 [2.052(6), 2.048(6) Å] and a deviation from linearity in the Zn1–C16–C17 angle to 155.8(5)° (*cf* 172.0(5) and 173.6(5)° for the comparable angles subtended at C8 and C24). The benzyl component of each zincate dianion does not interact with calcium and evidently arises from methyl activation of the toluene solvent. The resultant Zn1–C7 bond length of 2.059(6) Å is slightly shorter than in its closest comparator, *^t^* Bu_2_Zn­(PhCH_2_)­K·Me_6_TREN (2.109(4) Å), although in both instances a Zn-CH_2_–C angle of *ca.* 114° is observed.^64^

**Figure 6:**
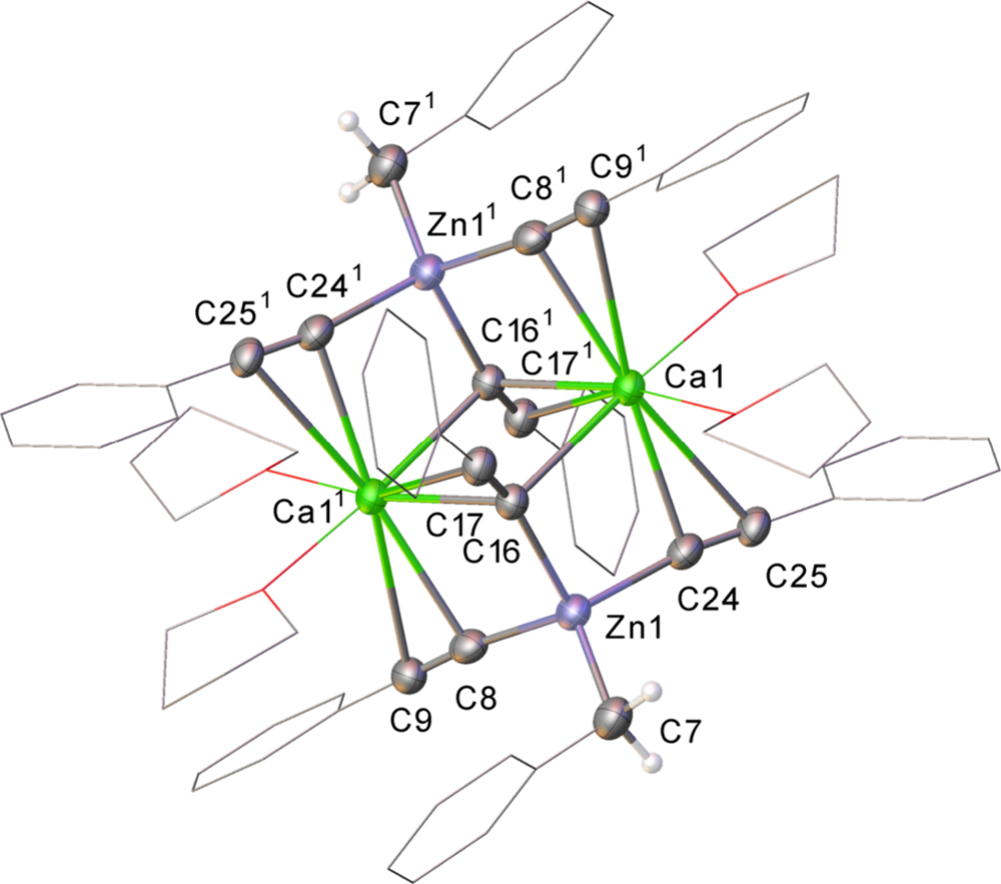
Molecular structure of **10**, displacement ellipsoids at 30%. For clarity, hydrogen atoms, apart from those on the benzyl CH_2_ are omitted. Dipp and phenyl groups are displayed as wireframe, also for visual ease. Selected bond lengths (Å): Ca1–Zn1 3.3208(12), Ca1–Zn1^1^ 3.3807(12), Ca–Ca1^1^ 4.0339(19), Zn1–Zn1^1^ 5.3517(14), Ca1–O1 2.371(5), Ca1–O2 2.359(16), Ca1–C16 2.648(5), Ca1–C16^1^ 2.643(5), Ca1–C17^1^ 3.117(6), Ca1–C24 2.626(5), Ca1–C25 3.041(5), Zn1–C16 2.128(5), Zn1–C24 2.048(5), Zn1–C8 2.051(6), Zn1–C7 2.059(6).

## Conclusions

In summary, treatment of [(BDI)­CaH]_2_ with [ZnTMP_2_] in the presence of terminal alkynes results in a sequence of calcium–zinc equilibria that are strongly dependent on substrate loading and identity. When two equivalents of alkyne are employed, the system initially converges on the formation of dialkynyl­(hydrido)­zincates, i.e. [(BDI)­Ca­(μ-C≡C*^n^* Bu)­(μ-TMP)­Zn­(μ-H)]_2_ and [(BDI)­Ca­(μ-C≡CPh)­(μ-TMP)­Zn­(μ-H)]_2_, which evolve through ligand redistribution and hydride reorganization to the corresponding β-diketiminato calcium acetylides. In contrast, when four equivalents of alkyne are introduced, complete TMP substitution can be achieved, affording the tetrakis­(alkynyl)­zincate species, [(BDI)­Ca­(μ-C≡C*^n^* Bu)_2_Zn­(μ-H)]_2_ and [(BDI)­Ca­(μ-C≡CPh)_2_Zn­(μ-C≡CPh)_2_Ca­(BDI)]. Overall, this study extends the scope of synergistic calcium–zinc chemistry to *sp* carbon fragments. We are continuing to explore the broader generality of this approach, with the aim of preparing further *s*-block heterobimetallic systems for bond activation reactivity.

## Experimental Section

### General Considerations

All manipulations were carried out using standard Schlenk line and glovebox techniques under an inert atmosphere of argon. NMR experiments were conducted in J-Young’s NMR tubes and prepared in a glovebox. NMR spectra were recorded on a Bruker BioSpin GmbH spectrometer operating at 400.13 MHz (^1^H) and 100.62 MHz (^13^C). Elemental analyses were performed at Elemental Microanalysis Ltd., Okehampton, Devon, UK or by the Elemental Analysis Services Team at London Metropolitan University. Solvents were dried by passage through a commercially available solvent purification system and stored under argon in ampules over 4 Å molecular sieves. C_6_D_6_, C_7_D_8_ and C_4_D_8_O were purchased from Merck, dried over potassium, distilled and stored over molecular sieves. 1-Hexyne, phenylacetylene, and 2-ethynylpyridine were dried over [CaH_2_]_n_, distilled and stored over molecular sieves for 12 h prior to use. [(BDI)­CaH]_2_ and [ZnTMP_2_] were synthesized according to literature procedures.^48,65^

#### Synthesis of [(BDI)­Ca­(μ-C≡C*^n^* Bu)­(μ-TMP)­Zn­(μ-H)]_2_ (**4**)

[(BDI)­CaH]_2_ (30 mg, 0.033 mmol) and [ZnTMP_2_] (22.8 mg, 0.066 mmol) were introduced into a vial and dissolved in *d*_8_-toluene (0.6 cm^3^). 1-Hexyne (7.6 μL, 0.066 mmol) was added and the solution was immediately placed in the freezer (−35 °C), affording colorless crystals after 16 h, identified as **4** through X-ray diffraction analysis. The supernatant was decanted, and the colorless crystals were washed with cold hexane (1 cm^3^). The crystals were crushed, and the hexane was allowed to slowly evaporate. Note: Applying vacuum to the crystals resulted in decomposition (BDI-H formation). Attempts to try and run the corresponding ^13^C NMR spectrum were unsuccessful due to slow conversion to [(BDI)­Ca­(μ-C≡C*^n^* Bu)]_2_, even at temperatures below −20 °C.

^1^H NMR (C_6_D_6_): δ = 7.12 (m, ^Dipp^Ar–H, 6H), 4.79 (s, HCCN, 1H), 3.80 (s, Zn–H, 1H), 3.35 (m, HC­(CH_3_)_2_, ^*3*^ *J*_*HH*_ = 6.75 Hz, 4H), 2.45 (t, C≡CCH_2_CH_2_CH_2_CH_3_, ^*3*^ *J*_*HH*_ = 7.04 Hz, 2H), 1.75 (s, NCCH_3_, 6H), 1.58–1.51 (m, N­(CH_3_)_2_{CH_2_}_2_CH_2,_ 6H), 1.42–1.40 (m, C≡CCH_2_CH_2_CH_2_CH_3_, 7H), 1.29–1.23 (overlapping doublets, HC­(CH_3_)_2_, 22 H), 1.18 (d, HC­(CH_3_)_2_, ^*3*^ *J*_*HH*_ = 6.75 Hz, 6 H), 1.07 (s, N­(CH_3_)_2_{CH_2_}_2_CH_2_, 12H), 0.95 (t, CCH_2_CH_2_CH_2_CH_3_, ^*3*^ *J*_*HH*_ = 7.16 Hz, 3H).

#### Reaction between [(BDI)­CaH]_2_ and [ZnTMP_2_] with 1-Hexyne

1-Hexyne (7.6 μL, 0.066 mmol) was added to a *d*_8_-toluene solution (0.6 cm^3^) of [(BDI)­CaH]_2_ (30 mg, 0.033 mmol) and [ZnTMP_2_] (22.8 mg, 0.066 mmol) inside a J. Young’s NMR tube. A single new BDI-containing species was observed spectroscopically (Figure S1) which attenuated over the course of 16 h, giving exclusive rise to [(BDI)­Ca­(μ-C≡C*^n^* Bu)]_2_ (Figure S3). [(BDI)­Ca­(μ-C≡C*^n^* Bu)]_2_ (**5**) was purified through a hexane recrystallization (Figure S4). Yield: 28 mg, 79%.

#### Reaction between [(BDI)­CaH]_2_ and [ZnTMP_2_] with Phenylacetylene

Phenylacetylene (7.3 μL, 0.066 mmol) was added to a *d*_8_-toluene solution (0.6 cm^3^) of [(BDI)­CaH]_2_ (30 mg, 0.033 mmol) and [ZnTMP_2_] (22.8 mg, 0.066 mmol) inside a J. Young’s NMR tube. A mixture of products was observed spectroscopically (Figure S6) alongside a significant amount of crystalline precipitate. The solid product was confirmed by X-ray diffraction to be [(BDI)­Ca­(μ-C≡CPh)]_2_, which, consistent with literature precedent, was insoluble in deuterated solvents in which it will not react.^62^

#### Reaction between [(BDI)­CaH]_2_ and [ZnTMP_2_] with 2-Ethynylpyridine

2-Ethynylpyridine (6.4 μL, 0.066 mmol) was added to a *d*_8_-toluene solution (0.6 cm^3^) of [(BDI)­CaH]_2_ (30 mg, 0.033 mmol) and [ZnTMP_2_] (22.8 mg, 0.066 mmol) inside a J. Young’s NMR tube, resulting in an immediate purple coloration. A single major BDI-containing species identified by NMR spectroscopy (Figure S7) was purified by crystallization at −35 °C, affording colorless crystals of **6** after 48 h, which were washed with cold (0 °C) toluene (1 cm^3^). Yield: 16 mg, 44%. Attempts to isolate zincate intermediates from this reaction were unsuccessful, providing only **6**, which can be made directly from [(BDI)­CaH]_2_ (*vide infra*).

#### Synthesis of [(BDI)­Ca­(μ-C≡CPy)]_2_ (**6**)

2-Ethynylpyridine (5.47 μL, 0.054 mmol) was added to a *d*_8_-toluene solution (0.6 cm^3^) of [(BDI)­CaH]_2_ (24.9 mg, 0.027 mmol), observing immediate effervescence. The solution was placed in the freezer, affording colorless crystals of **6** after 16 h. These crystals were washed with cold hexane. Yield: 25.4 mg, 84%. Placing **6** under vacuum results in decomposition.

^1^H NMR (C_7_D_8_): δ = 8.60 (d, C–N = CH-CH = CH–CH, ^*3*^ *J*_*HH*_ = 5.04 Hz, 1H), 6.84–6.77 (m, C–N = CH–CH = CH-CH, 2H), 6.42–6.39 (m, C–N = CH-CH=CH–CH, 1H), 4.84 (s, HCCN, 1H), 3.62 (m, HC­(CH_3_)_2_, 2H), 2.67 (m, HC­(CH_3_)_2_, 2H), 1.71 (s, NCCH_3_, 6H), 1.55 (br s, HC­(CH_3_)_2_, 6 H), 1.34 (br s, HC­(CH_3_)_2_, 6 H), 0.89 (br s with residual hexane signal on top, HC­(CH_3_)_2_, 6 H), 0.58 (br s, HC­(CH_3_)_2_, 6 H). ^13^C­{^1^H} NMR (C_6_D_6_): δ = 165.6 (NCCH_3_), 154.4 (C-N = CH–CH = CH–CH), 148.3 (C–N = CH–CH = CH–CH), 146.4 (*i-*C_6_H_3_), 145.0 (Ar–C), 124.5 (Ar–C), 123.3 (C–N = CH–CH = CH-CH), 121.5 (C–N = CH-CH = CH–CH), 111.2 (C≡C), 93.6 (HCCN), 28.8 (HC­(CH_3_)_2_), 24.7 (HC­(CH_3_)_2_), 23.5 (NCCH_3_). Anal. Calc. for C_71_H_87_N_6_Ca_2_: C, 77.20; H, 7.94; N, 7.61. Found: C, 76.67; H, 7.29; N, 3.23.

#### Synthesis of [(BDI)­Ca­(μ-C≡CPh)­(μ-TMP)­Zn­(μ-H)]_2_ (**7**)

[(BDI)­CaH]_2_ (30 mg, 0.033 mmol) and [ZnTMP_2_] (22.8 mg, 0.066 mmol) were introduced into a vial and dissolved in *d*_8_-toluene (0.6 cm^3^). Phenylacetylene (7.3 μL, 0.066 mmol) was added and the solution was immediately placed in the freezer (−35 °C), affording colorless crystals after 16 h that were identified as **7** by X-ray diffraction analysis. These crystals were extremely insoluble and any attempts to characterize **7** spectroscopically resulted in the formation of [(BDI)­Ca­(μ-C≡CPh)]_2_.

#### Synthesis of [(BDI)­Ca­(μ-C≡C*^n^* Bu)_2_Zn­(μ-H)]_2_ (**8**)

[(BDI)­CaH]_2_ (30 mg, 0.033 mmol) and [ZnTMP_2_] (22.8 mg, 0.066 mmol) were introduced into a vial and dissolved in *d*_8_-toluene (0.6 cm^3^). 1-Hexyne (15.2 μL, 0.12 mmol) was added and the solution was immediately placed in the freezer (−35 °C), affording colorless crystals after 16 h that were identified as **8** by X-ray diffraction analysis. These crystals were extremely insoluble and any attempts to characterize **8** spectroscopically resulted in the formation of [(BDI)­Ca­(μ-C≡C*^n^* Bu)]_2_.

#### Synthesis of [(BDI)­Ca­(μ-C≡CPh)_2_Zn­(μ-C≡CPh)_2_Ca­(BDI)] (**9**)

[(BDI)­CaH]_2_ (30 mg, 0.033 mmol) and [ZnTMP_2_] (22.8 mg, 0.066 mmol) were introduced into a vial and dissolved in *d*_8_-toluene (0.6 cm^3^). Phenylacetylene (14.6 μL, 0.12 mmol) was added and the solution was immediately placed in the freezer (−35 °C), affording colorless crystals after 16 h that were identified as **9** by X-ray diffraction analysis. These crystals were extremely insoluble and any attempts to characterize **9** spectroscopically resulted in the formation of [(BDI)­Ca­(μ-C≡CPh)]_2_.

#### Synthesis of [(THF)_2_Ca­(μ-C≡CPh)_3_Zn­(CH_2_Ph)]_2_ (**10**)

[(BDI)­CaH]_2_ (30 mg, 0.033 mmol) and [ZnTMP_2_] (22.8 mg, 0.066 mmol) were introduced into a J. Young’s NMR tube and dissolved in *d*_8_-toluene (0.6 cm^3^). 1-Hexyne (7.6 μL, 0.066 mmol) was added, the solution was agitated and phenylacetylene (7.3 μL, 0.066 mmol) was added resulting in an insoluble precipitate. This crude solid was not soluble in hydrocarbon solvents, therefore, a few drops of THF were added, resulting in dissolution and immediate precipitation/crystal formation. Only [(THF)_2_Ca­(μ-C≡CPh)_3_Zn­(CH_2_Ph)]_2_ (**10**) could be identified from this reaction by X-ray diffraction analysis.

## Supplementary Material



## Notes

NMR spectra and X-ray crystallographic details (PDF)
