# Supplementary material for: Calcium Alkynyl(hydrido)zincates
Source: Organometallics. 2026 Jul 9;45(14):1722–30. doi: 10.1021/acs.organomet.6c00164 (PMC13418192; doi:10.1021/acs.organomet.6c00164)
Supplement: Supplementary file 1 [file om6c00164_si_001.pdf]

Supplementary Information for

Calcium Alkynyl(hydrido)zincates

Marcos López-Aguilar,[ab] Kyle G. Pearce,*[a] and Michael S. Hill[a]

[a]*Department of Chemistry, University of Bath, Claverton Down, Bath, BA2 7AY, UK.*

[b]*Organic and Inorganic Chemistry Department, Instituto de Química Organometálica Enrique Moles, Universidad de Oviedo, 33006 Oviedo, Asturias, Spain.*

kgp29@bath.ac.uk

NMR Spectra

Figure S1-S4: NMR Spectra for **[(BDI)CaH]$_2$ + ZnTMP$_2$** with **1-Hexyne** **S2**

Figure S5: NMR Spectra for **[(BDI)Ca(μ-C≡C^{n}Bu)(μ-TMP)Zn(μ-H)]$_2$ (4)** **S4**

Figure S6: NMR Spectra for **[(BDI)CaH]$_2$ + ZnTMP$_2$** with **Phenylacetylene** **S4**

Figure S7: NMR Spectra for **[(BDI)CaH]$_2$ + ZnTMP$_2$** with **2-Ethynylpyridine** **S5**

Figure S8-S11: NMR Spectra for **[(BDI)Ca(μ-C≡CPy)]$_2$ (6)** **S5**

Crystallographic Data **S8**

References **S13**

NMR Spectra

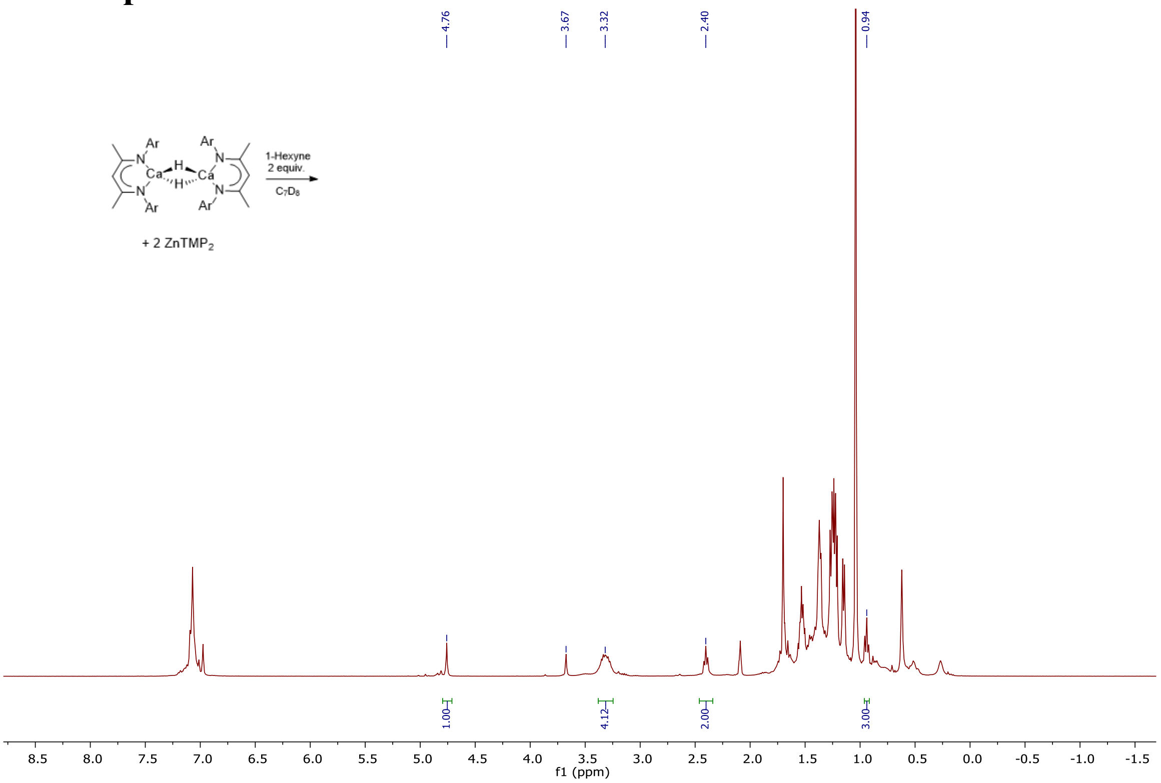

Figure S1. *^{1}H NMR Spectrum (C_7D_8, 298 K, 400.13 MHz) from the initial reaction of $[(BDI)CaH]_2$ + $ZnTMP_2$ + 1-hexyne.*

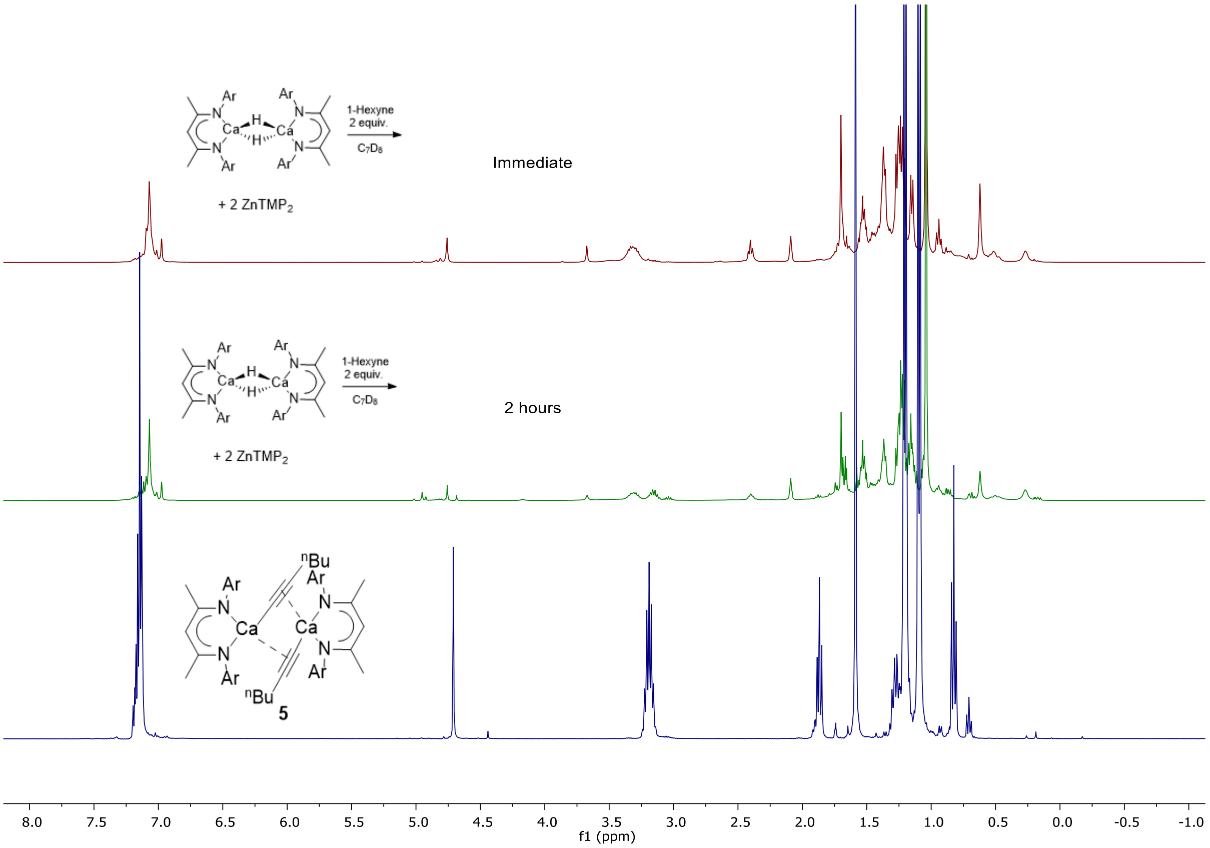

Figure S2. *^{1}H NMR stacked spectra (C_7D_8, 298 K, 400.13 MHz) displaying the spectrum from the reaction between $[(BDI)CaH]_2$ + $ZnTMP_2$ + 1-hexyne immediately and then after 2 hours. The spectrum of* ***[(BDI)Ca(μ-C≡C^{n}Bu)]$_2$*** *is also there for reference.*

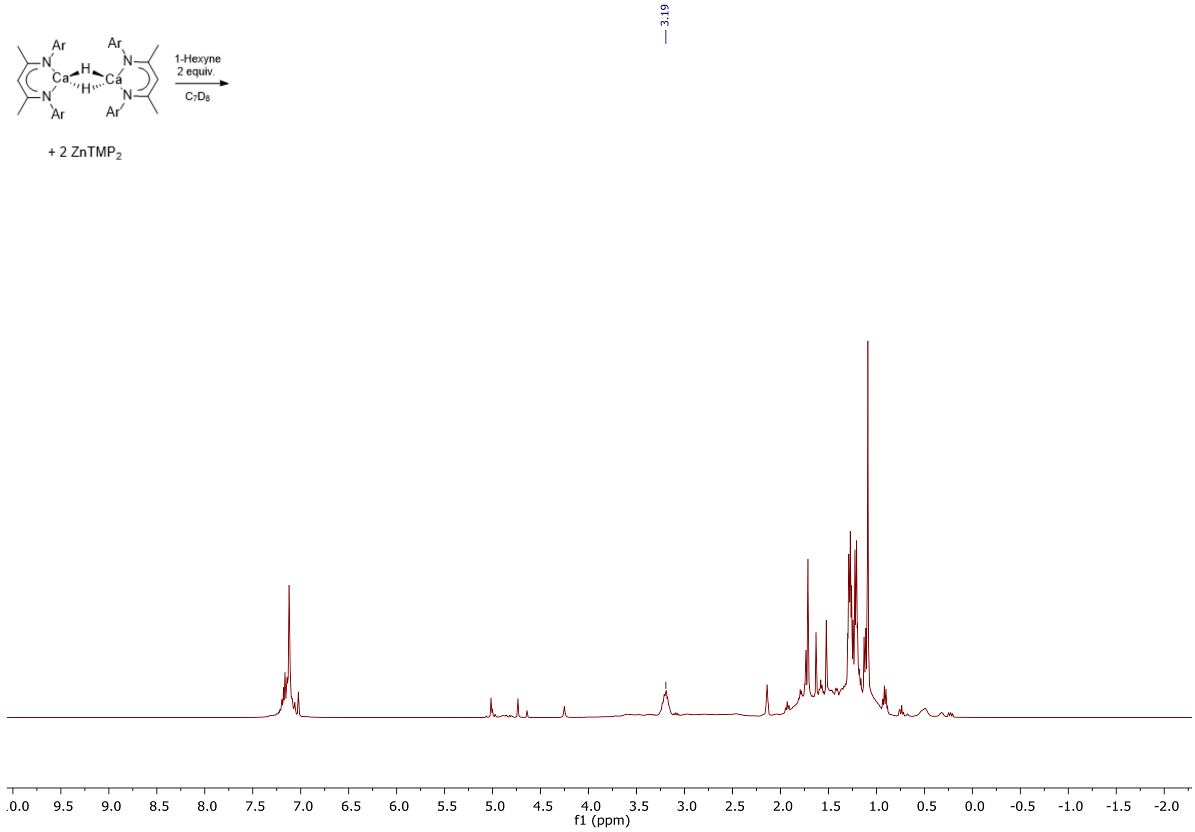

Figure S3. *^{1}H NMR Spectrum (C_7D_8, 298 K, 400.13 MHz) from the reaction of [(BDI)CaH]$_2$ + $ZnTMP_2$ + 1-hexyne after 16 hours.*

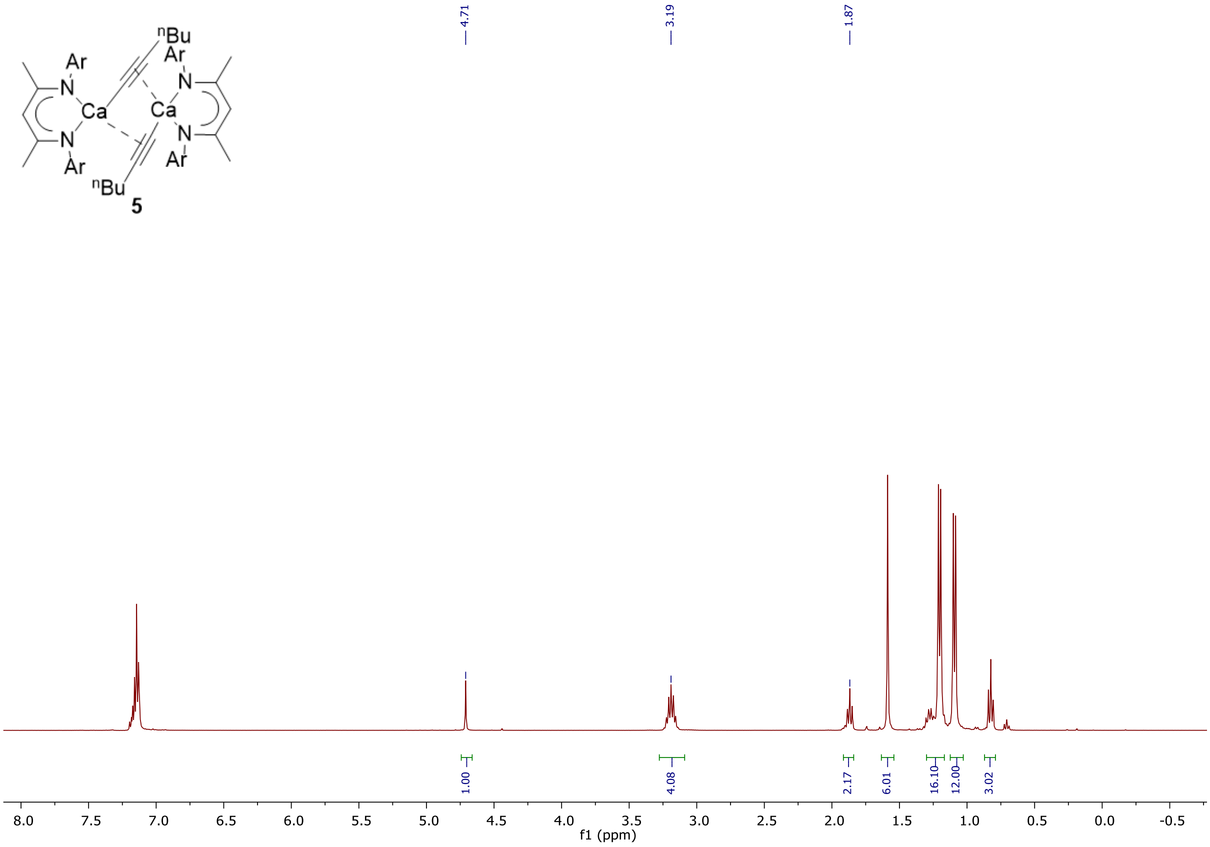

Figure S4. *^{1}H NMR Spectrum (C_6D_6, 298 K, 400.13 MHz) for recrystallised* ***[(BDI)Ca(μ-C≡C^{n}Bu)]$_2$****.*

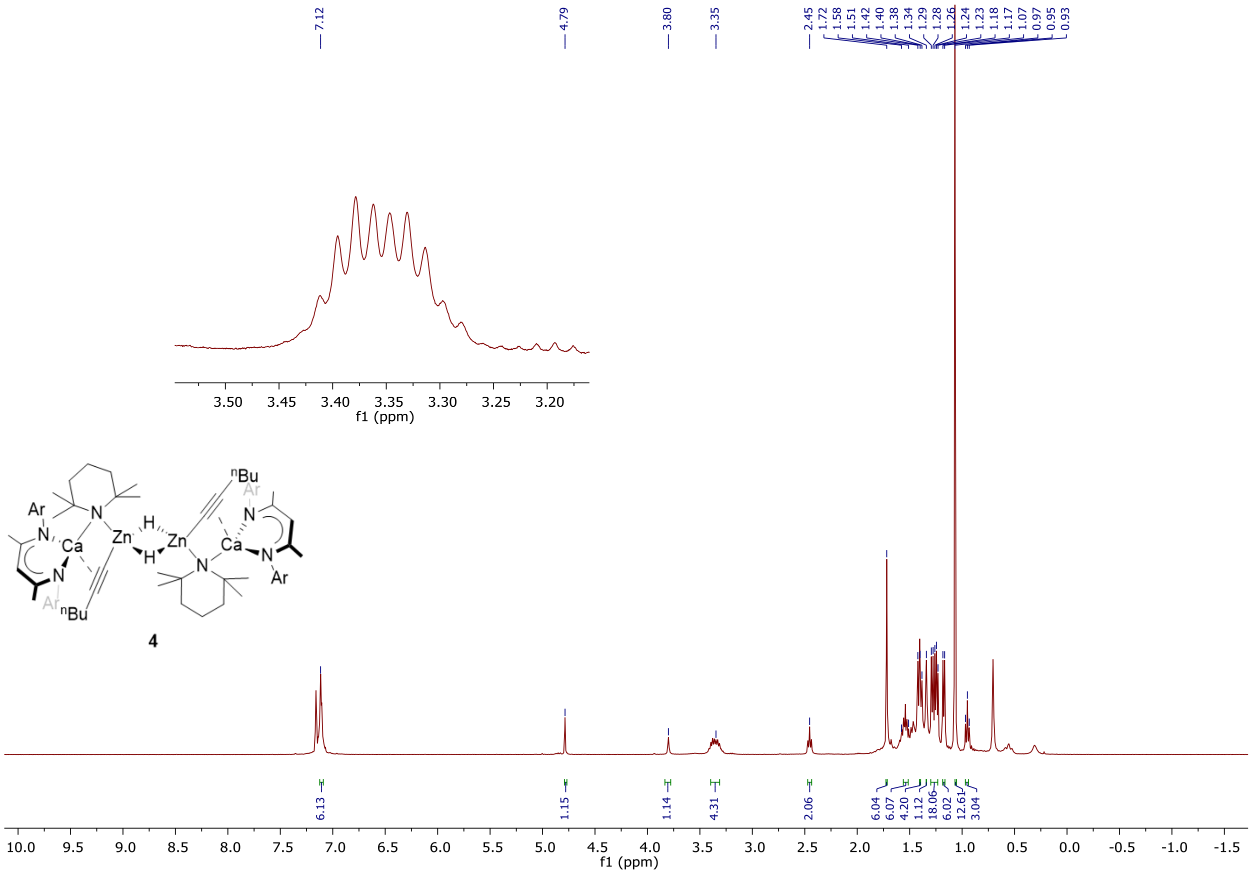

Figure S5. *^{1}H NMR Spectrum (C_6D_6, 298 K, 400.13 MHz) for* ***[(BDI)Ca(μ-C≡C^{n}Bu)(μ-TMP)Zn(μ-H)]$_2$*** *(4).*

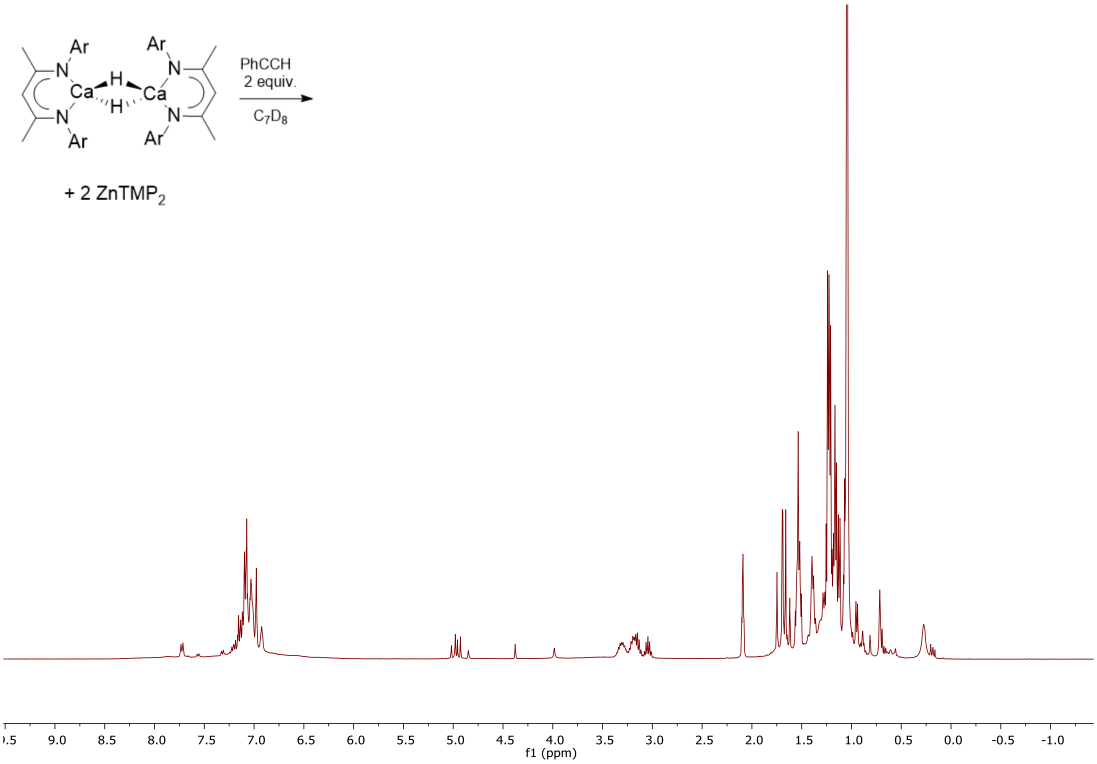

Figure S6. *^{1}H NMR Spectrum (C_7D_8, 298 K, 400.13 MHz) from the reaction of [(BDI)CaH]$_2$ + ZnTMP$_2$ + Phenylacetylene.*

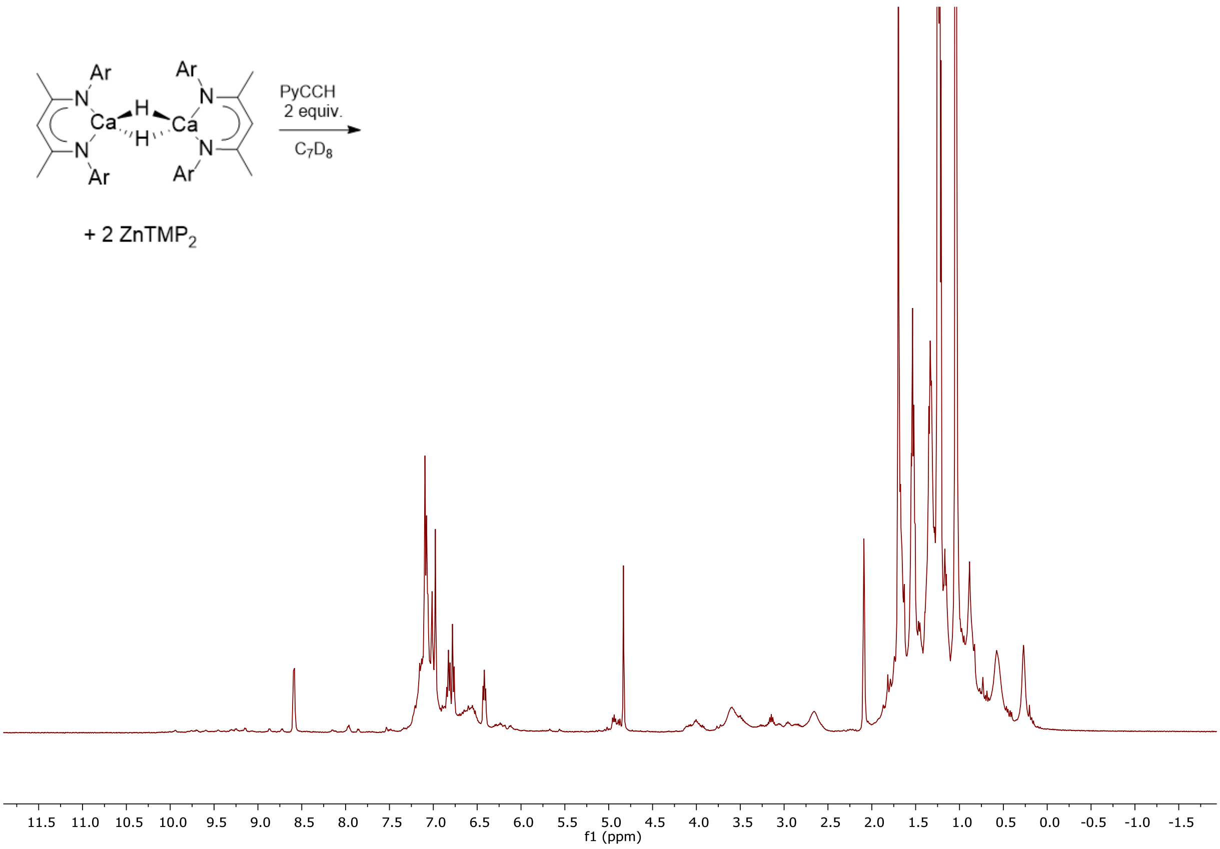

Figure S7. *^{1}H NMR Spectrum (C_7D_8, 298 K, 400.13 MHz) from the reaction of [(BDI)CaH]$_2$ + ZnTMP$_2$ + 2-ethynylpyridine.*

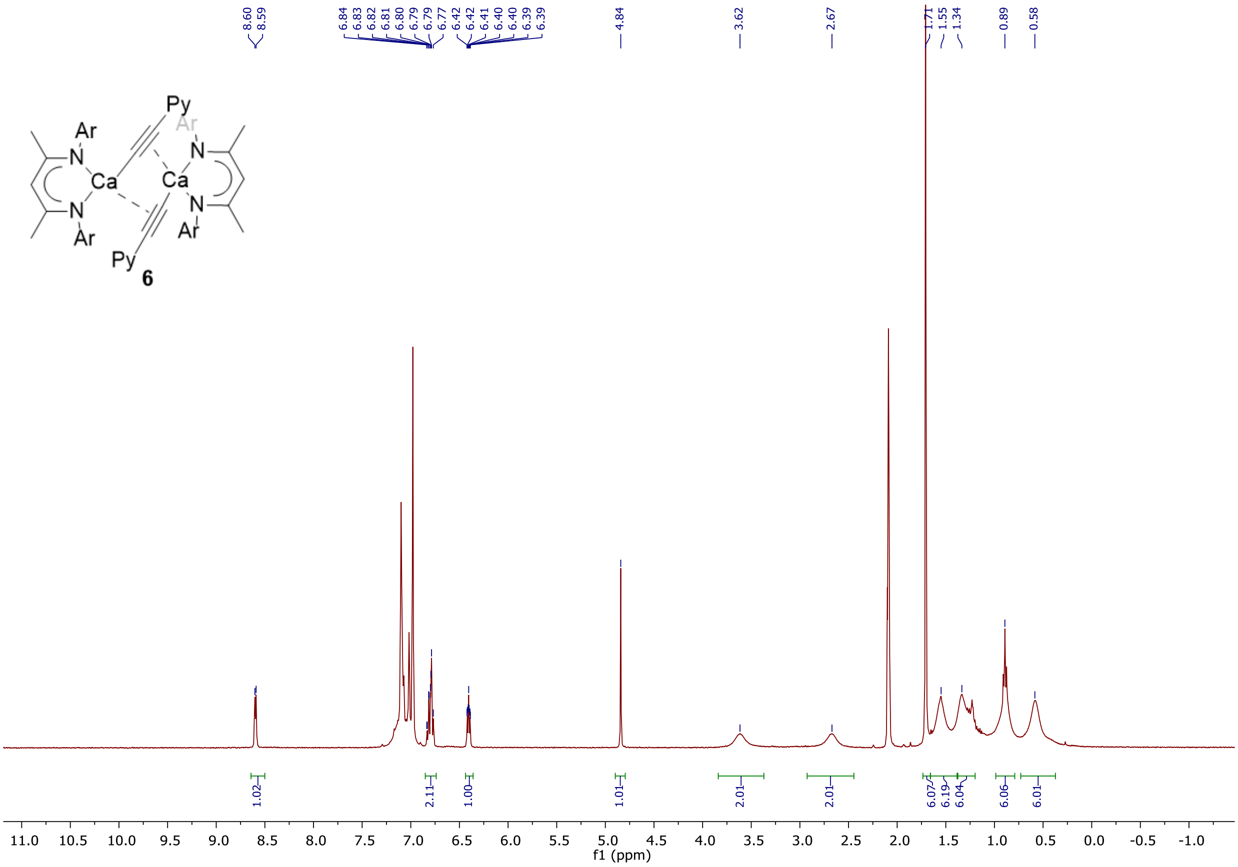

Figure S8. *^{1}H NMR Spectrum (C_6D_6, 298 K, 400.13 MHz) for* ***[(BDI)Ca(μ-C≡CPy)]$_2$ (6)****.*

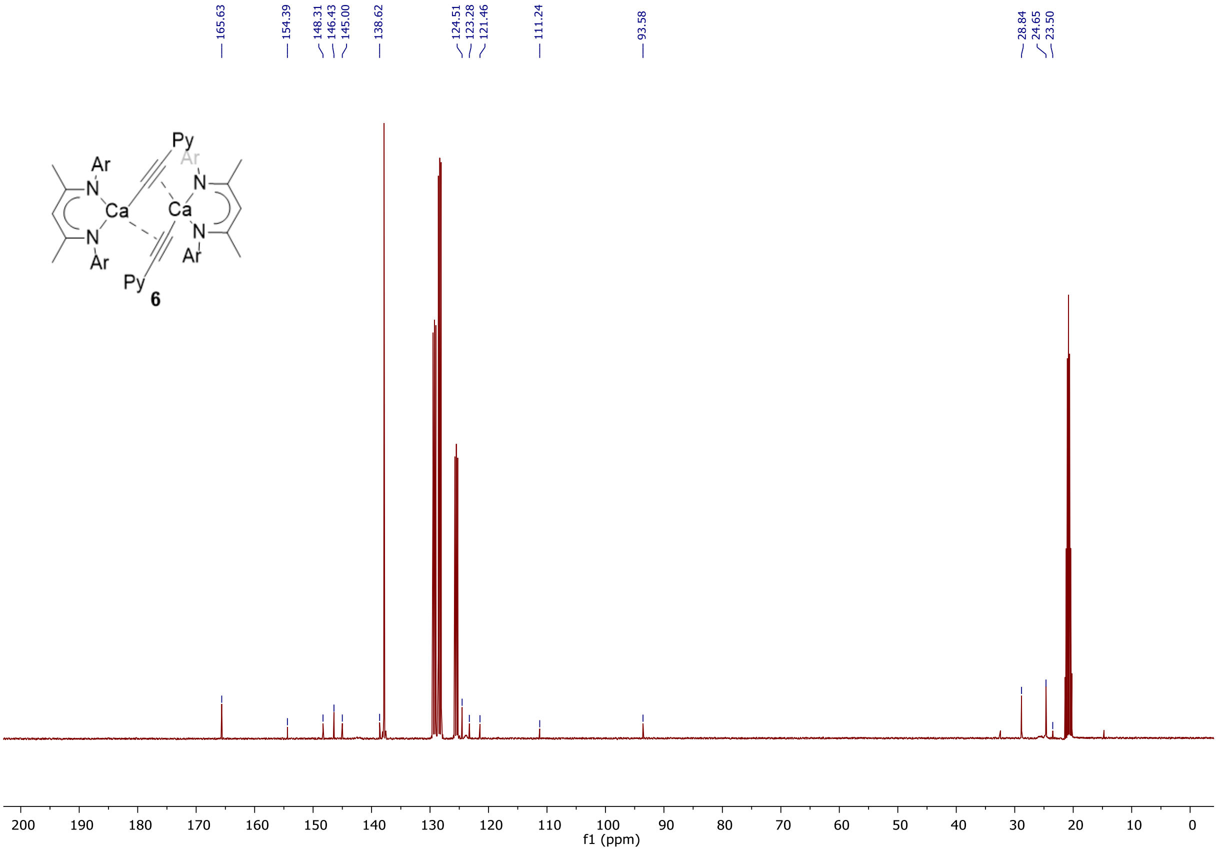

Figure S9. *$^{13}C\{^{1}H\}$ NMR Spectrum (C_6D_6, 298 K, 100.62 MHz) for* ***[(BDI)Ca(μ-C≡CPy)]$_2$ (6)***.

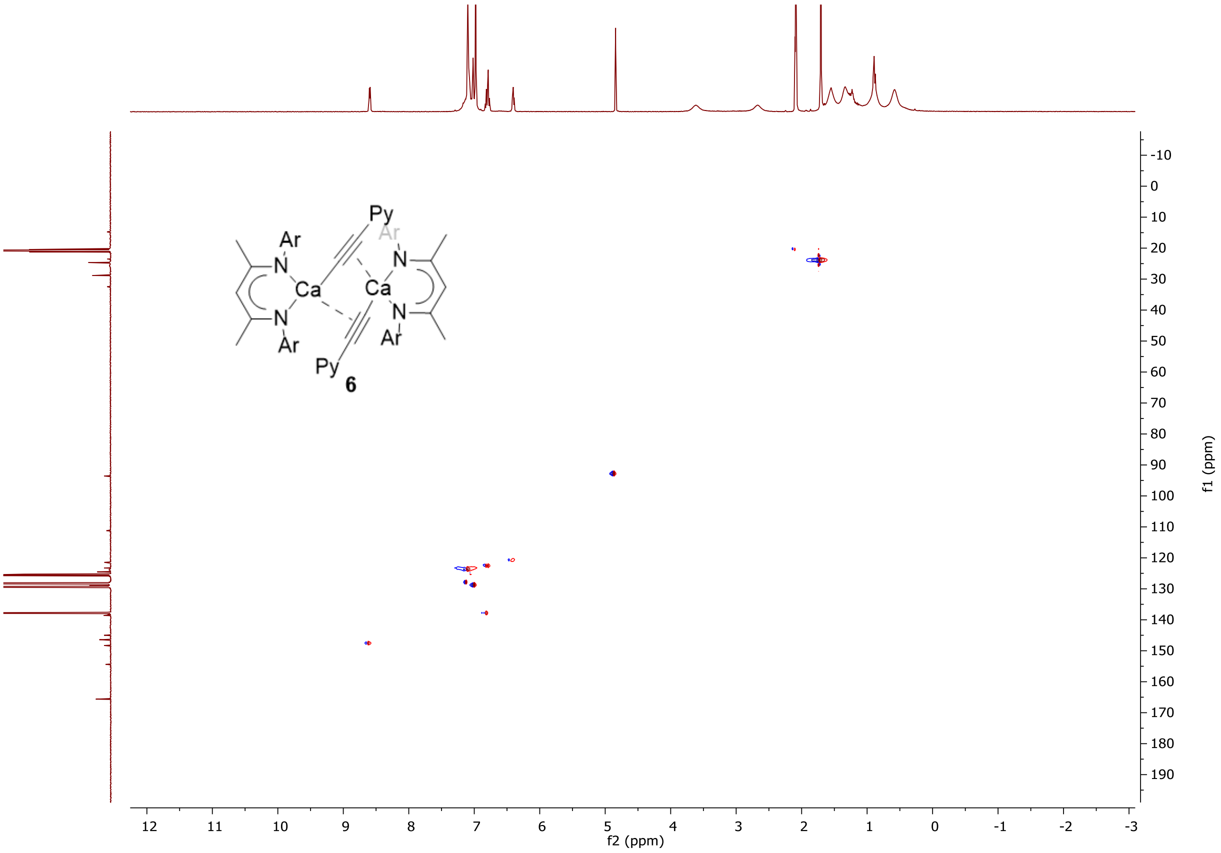

Figure S10. *^{1}H-^{13}C HSQC trace (C_7D_8, 298 K, 400.13, 100.62 MHz) for* ***[(BDI)Ca(μ-C≡CPy)]$_2$ (6)***.

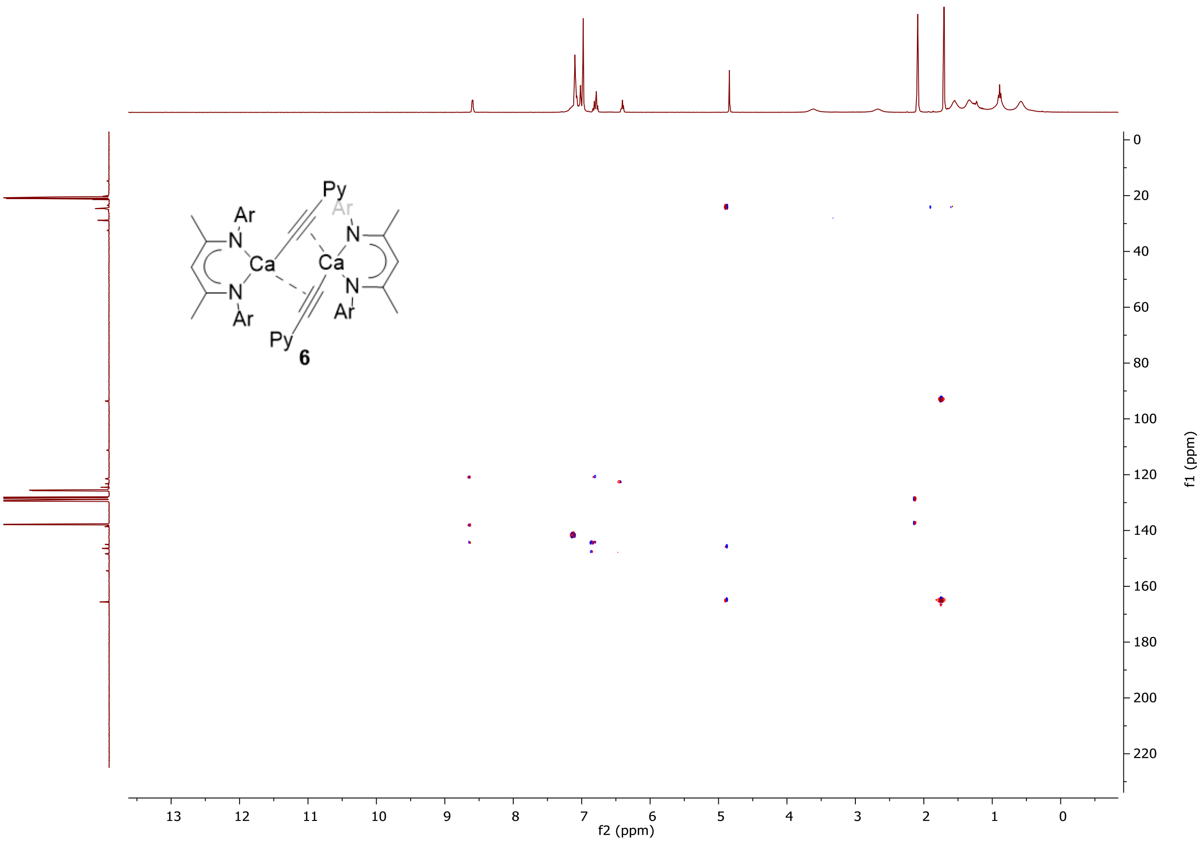

Figure S11. *^{1}H-^{13}C HMBC trace (C_7D_8, 298 K, 400.13, 100.62 MHz) for* ***[(BDI)Ca(μ-C≡CPy)]$_2$ (6)***.

Crystallographic Data

Single Crystal X-ray diffraction data for compounds **4** (s25msh47) and **5** (s21msh98) were collected on an Agilent SuperNova EosS2 diffractometer using Cu-Kα (1.54184 Å) radiation, whilst compounds **6** (y25msh65), **7** (y25msh72), **8** (y25msh63), **9** (y25msh64) and **10** (y25msh71) were collected on an XtaLAB Synergy, Dualflex, HyPix-Arc 100 diffractometer using Cu-Kα (1.54184 Å) radiation. In each case, the crystals were maintained at 150 K during data collection. Using Olex2,[1] the structures were solved with the olex2.solve[2] structure solution program or ShelXT and refined with the ShelXL[3] refinement package using Least−Squares minimisation.

The asymmetric unit in **s25msh47** (**4**) is comprised of half a molecule of a bimetallic zincate as well as half a hexyne molecule. All molecules can be completed by virtue of inversion symmetry. A solvent mask was implemented for the included free hexyne, accounting for 92 electrons per unit cell, consistent with the presence of 0.5 hexyne molecules per asymmetric unit. Disorder was identified for the three distal carbons on the hexyne unit (C33-C-35), which were readily modelled in a 75:25 ratio. Distance and ADP restraints were employed, on merit, to assist convergence.

The asymmetric unit in the structure of **s21msh98** (**5**) comprises half of a dimer and half of a benzene molecule. The remainder of each moiety arise *via* inversion centres that are intrinsic to the space group. C35 and C35 were modelled to take account of 60:40 disorder. Distance and ADP restraints were employed in this region of the electron density map, to assist convergence. Data were integrated to take account of sample twinning.

The asymmetric unit for **y25msh65** (**6**) is comprised of one half of a dimeric calcium acetylide as well as half of a toluene molecule, which can be completed through inversion symmetry: ^{1}1-X,1-Y,1-Z. A solvent mask was calculated and used for the toluene molecule, accounting for 100 electrons per unit cell, consistent with 0.5 toluene molecules per asymmetric unit.

The asymmetric unit for **y25msh72** (**7**) is comprised of half of a dimeric Zn-Ca bimetallic molecule, alongside a single molecule of toluene which resides upon a special position. All molecules can be completed by virtue of inversion symmetry. Two-fold disorder was identified for the toluene molecule, across the point of symmetry, and three of the Dipp isopropyl units. The disordered units were readily modelled in a 65:35 and 50:50 ratio, respectively, with ADP and distance restraints implemented to assist convergence.

The asymmetric unit for **y25msh63** (**8**) is comprised of half of a calcium-zincate dimer, alongside a molecule of toluene. The dimer molecule can be completed by virtue of inversion symmetry: ^{1}1-X,1-Y,1-Z.

The asymmetric unit in **y25msh64** (**9**) is comprised of a single molecule of a calcium zincate, as well as three molecules of toluene. Disorder was readily identified and modelled on two of the toluene molecules in a 45:55 (C98-C104) and 50:50 (C105-C111) ratio, respectively. Distance and ADP restraints were employed in the disordered solvent regions to assist convergence.

The asymmetric unit for **y25msh71** (**10**) is comprised of two half dimeric molecules, containing both Zn and Ca. Both molecules can be completed by virtue of inversion symmetry: ^{1}2-X,1-Y,1-Z; ^{2}1-X,-Y,2-Z. Two-fold disorder was identified on the aromatic component (C1-C6) of the benzyl unit in molecule 1 as well as the aromatic component (C65-C70) of the benzyl (C41-C46) moiety and one of the acetylides (C65-C70) on molecule 2 and across all four incorporated THF units (O1,C32-35; O2,C36-C39; O3,C71-C74; O4,C75-C78). These disordered components were modelled in a 65:35, 50:50, 50:50, 50:50, 50:50, 40:60 and 65:35 ratio, respectively. ADP and distance restraints were employed to assist convergence.

Crystallographic data for all compounds have been deposited with the Cambridge Crystallographic Data Centre as supplementary publications CCDC 2551629-2551635 for **6-10**, respectively. Copies of these data can be obtained free of charge on application to CCDC, 12 Union Road, Cambridge CB2 1EZ, UK [fax(+44) 1223 336033], e-mail: deposit@ccdc.cam.ac.uk.

Table S1. *Crystal Data and Structure Refinement for Compounds* ***4-6****.*

Identification code	s25msh47 (**4**)	s21msh98 (**5**)	y25msh65 (**6**)
Empirical formula	$C_{94}H_{148}Ca_2N_6Zn_2$	$C_{76}H_{106}Ca_2N_4$	$C_{79}H_{98}Ca_2N_6$
Formula weight	1573.08	1155.80	1211.79
Crystal system	monoclinic	triclinic	monoclinic
Space group	$P2_1/c$	P-1	$P2_1/n$
a/ Å	11.84460(10)	11.7088(3)	13.92860(10)
b/ Å	15.7833(2)	13.0556(5)	14.48310(10)
c/ Å	24.8024(3)	13.6436(5)	18.16360(10)
α/ °	90	96.015(3)	90
β/ °	101.0370(10)	111.453(3)	90.8910(10)
γ/ °	90	110.499(3)	90
U/ Å^3	4550.97(9)	1752.16(12)	3663.69(4)
Z	2	1	2
ρ_{calc}/ g cm^{-3}	1.148	1.095	1.098
μ/ mm^{-1}	1.967	1.723	1.682
F(000)	1708.0	630.0	1308.0
Crystal size/ mm^3	0.23 × 0.08 × 0.07	0.306 × 0.144 × 0.071	0.26 × 0.11 × 0.09
2θ range for data collection/°	6.674 to 146.702	7.224 to 146.536	7.808 to 160.14
Index ranges	$-10 \leq h \leq 14$, $-19 \leq k \leq 19$, $-30 \leq l \leq 30$	$-14 \leq h \leq 14$, $-16 \leq k \leq 14$, $-16 \leq l \leq 16$	$-17 \leq h \leq 17$, $-18 \leq k \leq 17$, $-23 \leq l \leq 22$
Reflections collected	62395	7337	53706
Independent reflections, R_{int}	9122 [R_{int} = 0.0518, R_{sigma} = 0.0281]	7337 [R_{sigma} = 0.0273]	7936 [0.0348, 0.0227]
Data/restraints/parameters	9122/21/497	7337/38/405	7936/0/375
Goodness–of–fit on F^2	1.036	0.980	1.055
Final $R1$, $wR2$ [I>=2σ(I)]	R_1 = 0.0320, wR_2 = 0.0785	R_1 = 0.0333, wR_2 = 0.0899	R_1 = 0.0430, wR_2 = 0.1164
Final $R1$, $wR2$ [all data]	R_1 = 0.0408, wR_2 = 0.0828	R_1 = 0.0392, wR_2 = 0.0919	R_1 = 0.0474, wR_2 = 0.1192
Largest diff. peak/hole/ e Å^{-3}	0.23/-0.31	0.41/-0.22	0.41/-0.30

Table S2. *Crystal Data and Structure Refinement for Compounds* ***7-9***.

Identification code	y25msh72 (**7**)	y25msh63 (**8**)	y25msh64 (**9**)
Empirical formula	$C_{106}H_{146}Ca_2N_6Zn_2$	$C_{96}H_{136}Ca_2N_4Zn_2$	$C_{111}H_{126}Ca_2N_4Zn$
Formula weight	1715.18	1556.98	1661.68
Crystal system	monoclinic	triclinic	monoclinic
Space group	$P2_1/n$	P-1	$P2_1/c$
a/ Å	14.1881(3)	12.66170(10)	18.62420(10)
b/ Å	20.2151(4)	13.47060(10)	25.7181(2)
c/ Å	16.7021(3)	14.49750(10)	20.84930(10)
α/ °	90	107.1070(10)	90
β/ °	90.347(2)	95.5300(10)	106.4480(10)
γ/ °	90	104.5210(10)	90
U/ Å^3	4790.31(16)	2248.80(3)	9577.71(11)
Z	2	1	4
ρ_{calc}/ g cm^{-3}	1.189	1.150	1.152
μ/ mm^{-1}	1.914	1.983	1.656
F(000)	1848.0	840.0	3560.0
Crystal size/ mm^3	0.12 × 0.07 × 0.05	0.27 × 0.23 × 0.16	0.21 × 0.12 × 0.06
2θ range for data collection/°	6.866 to 157.63	7.182 to 160.802	6.024 to 160.798
Index ranges	$-17 \leq h \leq 13$, $-25 \leq k \leq 20$, $-21 \leq l \leq 20$	$-13 \leq h \leq 16$, $-17 \leq k \leq 13$, $-18 \leq l \leq 18$	$-23 \leq h \leq 21$, $-32 \leq k \leq 32$, $-26 \leq l \leq 26$
Reflections collected	49666	36189	144023
Independent reflections, R_{int}	9808 [R_{int} = 0.0728, R_{sigma} = 0.0508]	9596 [R_{int} = 0.0285, R_{sigma} = 0.0253]	20647 [0.0385, 0.0219]
Data/restraints/parameters	9808/63/669	9596/0/474	20647/1279/1148
Goodness–of–fit on F^2	1.090	1.042	1.038
Final $R1$, $wR2$ [I>=2σ(I)]	R_1 = 0.0618, wR_2 = 0.1433	R_1 = 0.0497, wR_2 = 0.1397	R_1 = 0.0466, wR_2 = 0.1263
Final $R1$, $wR2$ [all data]	R_1 = 0.0783, wR_2 = 0.1499	R_1 = 0.0524, wR_2 = 0.1418	R_1 = 0.0578, wR_2 = 0.1346
Largest diff. peak/hole/ e Å^{-3}	0.47/-0.45	0.77/-0.48	0.72/-0.75

***Table S3**. Crystal Data and Structure Refinement for Compound **10**.*

Identification code	y24msh71 (**10**)
Empirical formula	$C_{39}H_{38}CaO_2Zn$
Formula weight	644.14
Crystal system	triclinic
Space group	P-1
a/ Å	12.3651(7)
b/ Å	16.4744(7)
c/ Å	17.5689(5)
α/ °	85.955(3)
β/ °	82.878(4)
γ/ °	68.382(5)
U/ $Å^3$	3300.3(3)
Z	4
ρ_{calc}/ g cm^{-3}	1.296
μ/ mm^{-1}	2.634
F(000)	1352.0
Crystal size/ mm^3	0.08 × 0.06 × 0.04
2θ range for data collection/°	7.582 to 153.096
Index ranges	$-14 \leq h \leq 15$,<br>$-20 \leq k \leq 15$,<br>$-21 \leq l \leq 21$
Reflections collected	40757
Independent reflections, R_{int}	12662 [0.0699, 0.0778]
Data/restraints/parameters	12662/652/976
Goodness–of–fit on F^2	1.040
Final *R*1, *wR*2 [*I*>=2σ(*I*)]	$R_1 = 0.0685$, $wR_2 = 0.1864$
Final *R*1, *wR*2 [all data]	$R_1 = 0.1386$, $wR_2 = 0.2265$
Largest diff. peak/hole/ e $Å^{-3}$	0.72/-0.75

References

1. O. V. Dolomanov, L. J. Bourhis, R. J. Gildea, J. A. K. Howard and H. Puschmann, *J. Appl. Cryst.,* 2009, **42**, 339-341.
2. G. M. Sheldrick, *Acta Cryst*., 2015, **A71**, 3-8.
3. G. M. Sheldrick, *Acta Cryst*., 2015, **C71**, 3-8.
